# The Complexities of Prescribing Assistive Equipment at the End of Life—Patient and Caregivers’ Perspectives

**DOI:** 10.3390/healthcare10061005

**Published:** 2022-05-29

**Authors:** Deidre D. Morgan, Eileen Willis, Kate Sweet, Pen Roe, Joana Rabaçal, David C. Currow

**Affiliations:** 1 Research Centre for Palliative Care Death and Dying, Flinders University, Bedford Park, SA 5042, Australia; 2 College of Nursing and Health Sciences, Flinders University, Bedford Park, SA 5042, Australia; eileen.willis@flinders.edu.au (E.W.); pen.roe@flinders.edu.au (P.R.); 3 Disability and Development Services, Department for Child Protection, Adelaide, SA 5000, Australia; kate.sweet2@sa.gov.au; 4 Hospital Egas Moniz, 1349-019 Lisbon, Portugal; joanarabacal@campus.ul.pt; 5 Faculty of Science, Medicine and Health, University of Wollongong, Wollongong, NSW 2522, Australia; dcurrow@uow.edu.au

**Keywords:** rehabilitation, assistive equipment, palliative care, occupational therapy, physiotherapy

## Abstract

Ongoing participation in valued and essential everyday activities remains a priority for people with advanced disease. This study sought to understand factors influencing patients with advanced disease and caregivers’ utilisation of assistive equipment that enable this participation. Employing a pragmatic approach, purposive sampling identified participants who were interviewed in their homes. A semi-structured interview guide was employed to elicit community dwelling patients’ and caregivers’ perspectives about assistive equipment utilisation. Recorded interviews were analysed inductively and themes were constructed from the data. Fourteen interviews were conducted with patients and caregivers. Patients had a range of cancers and COPD. Three empirically developed themes demonstrate the complexities associated with the use of assistive equipment at the end of life: 1. Enabling engagement in everyday activities; 2. Dependency—a two-way street; 3. The pragmatics of choosing, using or declining assistive equipment. Participants were motivated to use assistive equipment when it optimised their function, enabled participation and supported their values, roles and interests. Conversely, use of assistive equipment could be met with ambivalence as it represented deterioration or could cause conflict within relationships. Caregivers found assistive equipment made it easier for them to provide physical care. Skilled proactive assistive equipment prescription and training by allied health professionals enhanced patient and caregiver confidence and capacity to engage in everyday activities.

## 1. Introduction

People with advanced disease place high value on maintaining participation in everyday activities for as long as possible. This participation is associated with sustained quality of life [1,2,3,4]. Maintenance of full or part independence in everyday activities is an end-of-life priority, even when experiencing functional decline [5,6,7]. The importance of this participation is demonstrated in the Canadian Medical Assistance in Dying (MAiD) reports [8]. The most recent report states that the two top reasons cited by people with advanced disease for choosing MAiD, to end what they define as intolerable suffering, are the inability to participate in meaningful activities (84.9%) and/or dependency with self-care (81.7%). This highlights how critical participation in seemingly mundane activities is for people with advanced disease. The efficacy of symptom control for those with palliative care needs has improved over time, and when symptoms are better managed the importance of participation comes to the fore. Given that functional decline, dependency and decreased participation are inevitable for most people as they approach the end of life, and often for sustained periods of time, ways to mitigate this dependency warrants attention. Access to palliative rehabilitation and assistive equipment may serve to ameliorate some of this suffering and enable participation and weaken the desire to prematurely end one’s life [9,10].

Palliative rehabilitation, originally proposed > 40 years ago, aims to optimise function, provide emotional support and manage symptoms [11]. The World Health Organization (WHO) has now endorsed palliative rehabilitation as essential to the care of people with advanced disease [10]. They propose that “when integrated into palliative care, rehabilitation can slow decline and may reverse previous decline in physical and cognitive functioning…, prevent avoidable hospital admissions, length of stay and complication risk” (p. 21). One key rehabilitation approach for people with advanced disease is the skilled prescription and training in the use of assistive equipment (AE). Proactive provision of AE can help to optimise function and participation in everyday activities later in life. Assistive equipment in this context refers to pieces of equipment to enable patients with impaired function to better manage self-care and social activities [12]. Assistive equipment may be used by patients and their caregivers and can include, but are not limited to, shower stools, over-toilet frames, wheelchairs, gait aids and hoists or patient lifters. However, prescription of AE must be considered alongside patients’ and caregivers’ needs and priorities [9,10,13,14].

An increasing number of people receive palliative care in the community, supported by family and friends as caregivers [15]. Although the utilisation of AE can optimise the function and ease the care needs of people with advanced disease [16,17], the ongoing physical and emotional support of caregivers is often required for extended periods of time [18,19]. Provision of this care can also have significant physical, financial and social ramifications for informal caregivers [18,20,21]. This study sought to understand the perspectives of caregivers and community dwelling patients with advanced disease about their experiences using AE to help to optimise function and the impact of AE on caregiver physical burden.

## 2. Methods

***Study Design:*** This qualitative study employed a pragmatic approach [22] in which research methods were selected according to their relevance to the phenomenon under investigation, rather than adhering to a specific epistemology. A pragmatic worldview considers the nature of experience, the context in which experiences occur and the dynamic relationship between actions and consequences [23]. This pragmatic approach enabled the examination of the use of AE for those with the rapidly changing health status and fluctuating functional capacity of people with advanced disease [24].

***Sampling and recruitment:*** Purposive sampling was used to identify potential participants who were patients and caregivers receiving domiciliary rehabilitative care delivered by occupational therapists and physiotherapists with palliative care experience. Caregiver participants were partners or adult children providing live-in care to the patients. Eligibility criteria required participants to have a life-limiting illness or to be the caregiver of a person with a life-limiting illness. Ability to speak English and capacity to participate in an interview were also required. Nineteen potential participants meeting eligibility criteria were identified; however, four died prior to contact with one readmitted to hospital. The remaining fourteen patients and eleven of their caregivers were invited to participate in a face-to-face interview in a place of their choosing between August 2016 and December 2016. Cessation of recruitment was a pragmatic decision, influenced by funding and time availability. However, we note that a sample of 23 participants is an acceptable number for a qualitative study, answers the research questions and is consistent with a pragmatic methodology [25].

**Figure 1:**
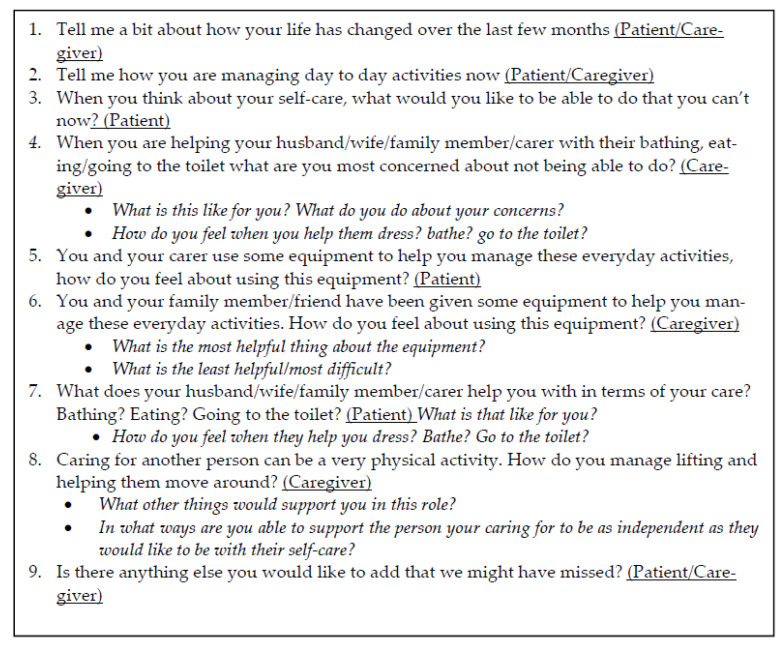
Interview guide.

***Data collection:*** A semi-structured interview guide, informed by clinical experience and pragmatic worldview, was employed to elicit patients’ and caregivers’ perspectives about AE (Figure 1). Interviews were conducted in the participants’ homes. No participants were known to the interviewers. Twelve one-off interviews were audio-recorded, lasting 10–60 min. Two interviews were not recorded (audio-recorder malfunction); however, notes were written up after the interview and included in the data analysis. Interviews were transcribed verbatim and participants ascribed a pseudonym. Transcripts were not sent to participants for member checking due to the advanced nature of their disease.

***Data analysis:*** NVivo software was used to organise and examine the data. A pragmatic inductive thematic analysis (DM, JR) enabled the iterative construction of codes and themes from the data [26]. Coding and themes were combined and a consensus on themes and sub-themes was reached by the full research team. Data have been reported against COREQ guidelines [27].

## 3. Results

A total of 14 interviews were conducted with patients and caregivers. Participant demographics are described in Table 1.

Three empirically developed themes demonstrate the complexities associated with the use of AE at the end of life (Table 2).

### 3.1. Enabling Engagement in Everyday Activities

Use of AE was experienced in a positive way when it helped to optimise independence and improved peoples’ confidence to participate in everyday activities.

#### 3.1.1. It’s Been Brilliant. I Can Get Out!

Each participant prioritised engagement in different types of everyday activities and a range of equipment enabled participation. Independent mobility inside the house and outdoor outings were highly valued and often only achieved with the use of a wheelchair or a walker. Wheelchairs and walkers enabled people to do essentials such as grocery shopping or attend social events such as the theatre, or a walk in the park with a partner.

**Kerry:** I use the wheelchair every Thursday when we go shopping because it’s too difficult for me to do every aisle in the shopping centre, not only with the cancer but I also have a chronic back… if I was walking… I’d have to stop for ten minutes and then walk for five, stop for ten. But in the wheelchair, we can do everything we want plus the other shops that I normally couldn’t.

**Anne:** If it is a nice day we will go and get a coffee and sit by the lake … and Dave (husband) will push me around if the weather is nice yeah or we just sit in the car but it’s fantastic.

Bed-poles and over-bed triangles reduced the effort patients needed to move from lying to sitting in bed. AE such as wheeled commode chairs, shower stools, grab rails and hand showers made it easier for people to manage changing toileting and showering needs.

**Adam:** Yeah, what I used to do was grab the sink on the vanity—grab the taps on the vanity and just give myself a bit of a pull… They also gave me a metal frame that goes around the toilet bowl with an arm rest…So when I sit on the toilet, my back’s straight, my knees are at 90 degrees, and I have got an arm rest and can get up and back on it and it’s very helpful.

**Christine:** I stand up, stick my head under, wash my hair and then I sit on the seat and wash the rest of myself… I will make sure someone is home and that Carl (husband) is home to help me if need be. He usually sets the chair up for me in the shower and then I have another chair that I sit on to dry myself.

While the use of a hospital bed complied with nurses’ manual handling requirements to assist with bathing in bed, for one couple, placing the bed in the lounge enabled the patient to be part of family life. She could lie down to sleep then adjust her position to sit up to watch television with her grandchildren, who would also climb on the bed with her to cuddle.

**Gillian—Husband:** Caroline would sleep a fair bit in the bed and I felt that if she had her bed out here at then if people came over—she can still hear because she’s got very good hearing—she doesn’t miss a thing, do you darling?

**Gillian:** Yeah.

**Gillian—Husband:** So then at least she is part of it and that I think was the important thing for me to have her within the lounge…being part of the whole family when they come.

Small portable threshold ramps enabled caregivers to lift commode chairs and wheelchairs over sliding door tracks or the lip of a shower recess with less physical effort.

**Paula:** …They made another step, the lower step, to go out through those back doors which has been really helpful, and I just have the little things over the sliding door tracks have been good so there’s nothing that we really haven’t used, have we?

Modification of existing steps to include additional shallower steps that were wide enough to accommodate a walking frame, made it less effortful for participants to descend and ascend the steps. This enabled participants to get outside independently, which was a valued part of regular routines.

#### 3.1.2. I Feel More Secure and Confident

Participants described the capacity of AE to mitigate and manage the impact of symptoms such as fatigue, weakness and breathlessness. They reported fear of falling in the shower because of growing leg weakness or wobbly legs which was eased with the use of a shower stool.

**Interviewer:** So, what is the most useful thing about the shower seat for you?

**Jane:** Just the fact that it is there when I need it or when I am not doing the best and I am feeling a little bit wobbly then—I have good days and bad days as well.

Handrails in the shower also helped provide stability, fostered confidence and enabled participants to shower while standing, something most preferred to do for as long as possible.

**Mark:** If I didn’t have the handrails, I would probably be just sitting in a chair instead of standing up in the shower; washing and do all that.

Another participant described how she relied on her husband and son to physically lift her off the toilet until she borrowed her sister’s over-toilet frame. The extra height meant she did not have to exert as much strength and was able to stand independently from the toilet.

**Christine:** [I was] getting stuck on the toilet… because the seat’s low. I’ve got no strength in my arms or my legs much so I couldn’t sort of lever myself up and get up

This was echoed by several participants.

**Mark—Wife:** But one of his favourite things is the toilet seat

**Mark:** Oh yeah! The raised toilet seat… Just like a donut, yeah, it’s brilliant…You’re just higher so when you want to stand up your legs aren’t bent. I reckon they should have them in every household. They should be built with them because when you go to stand up you don’t really have to lift your whole weight because your legs are up there.

Gait aids and shower stools helped patients and caregivers to manage the fatigue they experienced during everyday activities.

**Kerry:** If it’s a full day out or a full shop, I will take the wheelchair because it just drags it out of you. … If I’m feeling really alert and know that I’ve got a little bit more energy, I will go without [stick], but if I haven’t and I feel blah, I will use the stick and just take a little bit more time, but either way it still drags it out of me… I can still do a lot of things, it’s just the fatigue and no strength is what stops me at the moment.

Gait aids improved participants’ ability to ambulate, increased their sense of security against falls, provided stability and enabled them to walk longer distances.

**Derek:** It gives you a little bit of... not satisfaction it gives you, it gives you a bit of protection been though it might not you feel as though it does.

**Interviewer:** Protection?

**Derek:** Against a fall.

Wheelchairs enabled participants to manage fatigue and weakness but were also used to manage breathlessness and enabled social outings.

**Frank-Daughter:** We had to hire one like for taking him out at night and it’s simply the transport for me of wheeling, you know, oxygen bottles and stuff like that. But we have a pride issue with wheelchairs, which I get…

**Frank:** …but it’s like Maree (daughter) said she’s got to hook the bottle on the cart there dragging the golf buggy around.

### 3.2. Dependency: A Two-Way Street

Participants and caregivers were cognisant of the demands of increasing dependency and managed this in different ways. AE impacted relationships and the presence or absence of support in its use could enhance or compromise caregiver confidence.

#### 3.2.1. Minimising Dependency on Caregivers

Patients were deeply appreciative of the support from caregivers. However, patients and caregivers were cognisant of the impact of this physical assistance on caregiver wellbeing and that remaining at home was likely contingent upon caregiver health. Patients did not want to be a burden on caregivers and caregivers hoped they could continue to physically manage care provision. Participants expressed concern about caregivers lifting heavy, awkward wheelchairs and lifting walkers in and out of car boots. One couple bought a new car and sourced a lighter wheelchair to ease the demands of physical lifting.

**Anne:** That’s the only thing that worries you is what happens when his (husband) health doesn’t get—if anything happens to him or if he hurts his back or something but at the moment, he’s good… We have got a wheelchair in the garage that belonged to Dave’s mum but it’s got big wheels and it is quite bulky and it’s a bit heavier to move and we used to have that before we got the other but the other one’s much lighter to move.

AE could be unwieldy to move and take some adjusting to.

**Paula—Husband:** I mean if the house was a bit bigger it would be easier to move—I put a few hits on the wall. I came around with a wheelchair and I smashed a plug that was in [it]…I smashed that to smithereens.

However, AE could also eliminate the need for a caregiver to carry a loved one or make the physical caring role less effortful with personal care or social activities.

**Paula—Husband:** Once I put her on the commode then I could put her over the toilet or put her in the shower… It had the wheels on the bottom and Dom Care also provided ramps into the shower and ramps into the house, you know what I mean… so I didn’t have to carry her.

Although AE could make it physically easier for a patient or caregiver to manage everyday activities, dependency on the use of AE could also exacerbate existing relationship tensions or create new ones. One participant described how she chose not to accept items of AE because her husband would become moody if she didn’t physically rely on him for care. Another participant chose to use a wheelchair or a walking stick for grocery shopping depending on her husband’s mood.

**Kerry:** Well, that’s due to relationship problems, mental fatigue, but yeah it also depends on what mood my husband’s in too, as whether we can get in and out within five minutes, or he’s in a good mood and we can be 15/20 min.

Loss of independence and reliance on a caregiver to assist with wheelchair mobility could be disconcerting and required open conversation and negotiation.

**Margaret—Daughter:** When we go out in the wheelchair, yeah. I think it’s easier for mum-no let me rephrase it, it was more comfortable for mum to be in the wheelchair rather than trying to walk, but somebody else steering, driving, pushing, stopping, starting with mum not in control… and you were worried about crossing roads.

**Margaret:** Yes, it seemed as if, it seemed as if they’ve (daughter) pushed me in front of a car, and [okay], and then waited, you know I was the one who was going to get cleaned up with the car. In a wheelchair so that you’re sitting sort of radiator height of the car coming to you. And, it is, I’ll say a bit of a concern.

#### 3.2.2. Ways That Caregivers Manage Dependency

Caregivers expressed concern about the physical impact of caring on their health, their partner’s wellbeing, their future capacity to care and desire to avoid injury.

**Frank—Daughter:** I’m not physically big. It’s getting him in and out the car and I know how hard that was when he wasn’t well…. If he goes down, I’m going down…I don’t want to deprive him of going out just because I’m not physically capable of getting him in and out of the car, whereas if I’ve got a wheelchair … at least I know that he can still enjoy the things that he enjoys without putting a physical strain on me.

**Gillian—Husband:** I was worried that—I am pleased we have got the stool and the shower the way we did because before I was trying to do it on my own … we were struggling, fear of a fall, and… with a stool it’s great because I could actually move in the shower as well if I needed to. I don’t like to lift her or anything like that just in case I do it wrong and I don’t want to hurt myself too...’No, no good to anybody—I’ve gotta be careful.

When patients’ health and function deteriorated, caregivers found the use of adjustable height AE invaluable to minimise lifting and to enable positioning for care.

**Paula—Husband:** That’s great, that’s great because you can lift her up—I can move the bed forward, move the bed up, move the bed. Before … [it] was very difficult for her because as I lifted her up, I was putting pressure on her so, now she stays in bed, she’s not moving so it’s better for her.

However, while AE eased the physical demands of caring, caregivers were not always trained in its use, especially if it was acquired informally from family or friends. When they did receive training about correct manual handling techniques with walkers and wheelchairs from occupational therapists and physiotherapists, they found that it increased their confidence and eased the physicality of caring.

**Margaret—Daughter:** And somebody said “Don’t do that, this is how you walk up the steps with a walker.” And she just went, see there… lift it up, take a step forward, move it up the step. God, I wish somebody had told us that when we first got it…. We were trying to work out the steps into the house and the steps out of the house… I was struggling.

**Anne:** The only experience I have had with a walker was my husband’s mother, she had one, and she got-she used to let it get away from her and she looked so bent over and we always used to say to her stand up straight. —You see so many people with walkers and they don’t know how to use them properly and I was so grateful that the physio came out here and showed me how to use it.

### 3.3. The Pragmatics of Choosing, Using or Declining AE

A number of factors contributed to participants’ decisions to use or ignore AE. Some adopted a pragmatic approach based on present or anticipated future needs or what was available. Choices were also influenced by perceptions around age appropriateness or environmental constraints.

#### 3.3.1. If You Need It, You Need It

A recurrent theme influencing participant acceptance and use of AE was that of pragmatism—simply put, “if you need it, you need it” ***(Kerry).***

**Adam:** there might have been a time where I might have thought—walking frame, I don’t need the toilet raised, I don’t need a frame around the toilet but I am realistic, I do need it and I am grateful that I’ve got it…It serves it purpose, it does what it is intended to do and it is very helpful.

**Christine:** That’s going to help me so much… so that didn’t bother me at all. All I could see was all the help that you know you people were going to give me… I don’t care, as long as it helps me and I can still get around.

Participants used equipment because it helped them to feel safer doing everyday activities and to maintain independence for longer.

**Anne:** It has changed dramatically in the last few months. When the equipment came—wheelchair, walker, shower chair and got the toilet seat—I thought I won’t need this walker. I’ve lost all the power in my legs so this has been invaluable—if I hadn’t had that I wouldn’t be able to get around.

**Christine:** I don’t sort of want anybody—I don’t want to be relying on anybody sort of thing… I would like to stay as independent as I can for as long as I can.

Access to and choice about what AE participants might use were largely contingent upon what was readily available from health professionals, family or friends. Participants described taking equipment, even if it was not needed at the time, just in case they might need it later.

**Adam:** I was given advice that if domiciliary care offer you any equipment, take it, because if you don’t take it and a few months down track you find you could have done with it, you might have trouble getting it. So, I took it—it was there on offer, I said ‘okay. we’ll have it’…It’s quite possible because I am not getting any stronger so there might be a time when I do need it but at the moment, I don’t feel that I need it but it’s there if I do.

Others made do with what they could get from hardware stores or other sources to keep doing what was important. One participant, a keen fisherman, acquired a wheelchair and paid a man to build a wheelchair ‘tow bar’. To this he attached a converted overbed table, a “trailer” and loaded this with his fishing rods and walker. His son pushed all this, and his father, down to the jetty to fish “every time the weather is nice.”

**Derek:** So, what we do then is put the wheelchair in behind that, put your [gear] on the trailer, and with the walker, that goes in behind the wheelchair with the fishing rods on it … and you got one bloke (son) pushing all the stuff there.

#### 3.3.2. If I am Not That Old Yet… Maybe One Day

Although AE could be welcomed by participants, this was not always the case. A recurrent theme was that of not wanting equipment now, although acknowledging they might need it later. Some considered AE something older people used and not appropriate for them, embarrassing or just something they would rather do without.

**Interviewer:** And you are not keen on the walker? Can you talk about why?

**Gillian:** I am not that old yet.

**Margaret:** Karen (daughter) told me that they put a pole in I thought ‘Oh god!” …Because I’m not old and I didn’t want a stick to get out of bed with you know.

Some participants simply did not wish to use AE or declined it before seeing it as they could not picture how it might help. Others considered AE to be more of a burden than assistance or their physical needs fluctuated and they did not need it every day.

**Kerry—Patient:** Sometimes it’s more of a burden to actually have it. It’s easier just to hold onto a shopping trolley and walk behind a shopping trolley than actually physically have to use the stick.

Equipment was described as problematic when it was too big for a bathroom or too heavy to move about. When patients felt using equipment could lead to mess such as urine splashback from sitting high on an over-toilet frame, they chose not to use it. One participant could not control the direction of his urine into a urine bottle, so he abandoned it, using a commode instead.

**Frank—Daughter:** We got the bottle because we thought it would be a good idea and Dad was always conscious that…he was knocking it over or the stream [sprayed out]. So he actually reverted to peeing in the rubbish bin, because it was easier to place it, wasn’t it? But then he’d get wee on his pyjama pants… So we tried a multitude of things prior to actually getting the commode…It’s been brilliant for him.

One participant wanted a rail for security and independence in her shower over the bath but lived in a rental property and the landlord would not approve any alterations. Utilisation of portable options such as a clamp rail was not possible due to the longstanding disrepair of the bathroom.

**Kerry:** I just have to have someone to help me get a—because our bathroom is falling apart and we’re waiting to get a new one. I have to have someone there who I can hang on to because there’s nothing to hang on to…. It’s been like that for three and a half years… and she’s [real estate agent] fighting with him to get everything fixed and he won’t do it.

## 4. Discussion

Findings demonstrate the complexities of using AE at the end of life from patients’ and caregivers’ perspectives. AE provided by occupational therapists and physiotherapists supported participants’ adaptation to deteriorating function and symptom burden [11,17,28,29]. Consistent with emerging research that highlights the importance of participating in essential and valued activities at the end of life, some participants prioritised management of self-care, while others prioritised social outings, describing how AE enabled them to participate in leisure activities. Motivation to use or decline AE can be examined through a pragmatic paradigm and also through the Model of Human Occupation (MOHO), an empirically developed occupational therapy theory. Both pragmatism and MOHO consider the dynamic systems in which people interact with each other and their environment. MOHO postulates that participation is patterned by a person’s sense of self-efficacy and capacity (volition), habits, roles and values within one’s environment [30]. Volition, as defined by MOHO, is influenced by personal causation (self-efficacy and self-capacity), values and interests. Study participants were more motivated to accept and use AE when it supported their sense of self-capacity and self-efficacy which is consistent with research with other cohorts, earlier in the disease trajectory. This was described by patients and caregivers alike in terms of independence, reduced effort moving, confidence and feeling secure [12,31,32,33]. However, utilisation of AE was also associated with ambivalence when it compromised values, roles and interests. Equipment was abandoned when its use created tension or was perceived to undermine roles and relationships with family members, was a reminder of deterioration or was simply too awkward to use [12,31,32,33,34,35,36]. Although evidence demonstrates palliative rehabilitation can improve or maintain participation through proactive skilled AE prescription and training [9,10,28,37], its value and efficacy are not always well understood [38,39]. Study findings shed further light on this.

In this study, prescription of AE was perceived to attenuate the impact of symptoms on function and support participants to manage everyday activities [9,12,16,40,41,42,43]. However, equipment prescription requires skilled assessment of patient needs, an understanding of disease trajectory and symptom burden, current and future caregivers’ physical, cognitive and emotional capabilities and the equipment solutions best suited to the situation [44]. When training in the use of AE was provided from occupational therapists and physiotherapists (a rehabilitative approach), this improved caregiver confidence and patient function. However, when equipment was sourced from family or friends, without training in its use, caregivers were less confident. Skilled proactive and timely AE prescription and training is critical to help optimise the function of people with advanced disease [31,36,41,45,46,47], as is the best match of equipment to patients’ diagnoses and expected disease trajectories [36,41,48]. People with advanced disease may experience rapid changes and fluctuations in their functional capacity requiring continuous evaluation of equipment and training needs [44]. Regular review of AE is recommended to ensure its appropriateness as patient function and caregiver capacity to use it changes [36,47,48]. Although the WHO emphasises the importance of timely access to appropriate AE [10], this can be complicated for people at the end of life by continuously changing funding schemes influenced by prognosis, health insurance and variable access to allied health professionals with appropriate expertise regarding the most suitable AE equipment [31,49,50].

Participants described dependency on AE and each other in the patient/caregiving relationship. Patients were concerned about being a physical burden [51], while caregivers expressed concern about not managing physical care or hurting their loved one [18]. Although physical caregiver health is a significant issue, with caregivers often sustaining physical injuries themselves [52], palliative care research into caregiver burden focuses predominantly on psychological distress [53,54,55] with minimal attention being paid to the physical burden of caring and the mitigating effect of AE on this experience [56]. Our findings build on earlier research on disease trajectory in which the use of AE helped to optimise independence, and improved caregiver confidence about managing the physical aspects of care to enable participation more safely [50]. Provision of AE together with training can enhance caregiver self-efficacy and self-capacity to manage physical care [30], which has also been found to support participation, a priority for people with advanced disease [12].

***Limitations and areas for future research:*** This study examined the utilisation of AE by patients with advanced disease and their caregivers. All caregivers, with one exception, were interviewed in the presence of the person for whom they were caring. Caregivers may have been more open about difficulties utilising AE and managing care if interviewed alone [57]. There is a paucity of research on the physical impact of caring and potential injuries caregivers may sustain. A small Australian study found that 43% of caregivers sustained physical injuries whilst providing care and required additional assistance to continue caregiving [52]. Utilising AE to maintain caregiver physical health has a significant role to play in reducing secondary health service utilisation by caregivers and requires quantification. Five interdependent stages have been identified when adjusting to AE (*evaluating* and *acknowledging* needs, *incorporating* and *using* AE, *future use*) [58]. Adjustment is noted to take time, something our participants did not have. Future research could examine factors influencing adjustment to AE in compressed end-of-life time frames. AE in this study was provided by allied health professionals experienced in working with people with advanced disease; however, not all allied health professionals feel confident working with this cohort [59]. Future research could examine factors associated with AE prescription by allied health professionals with limited experience in palliative care.

## 5. Conclusions

Utilisation of assistive equipment by community-dwelling palliative care patients and their caregivers is influenced by a range of factors. Participants were motivated to use AE when it helped to optimise function, enabled participation and supported their values, roles and interests. Caregivers found AE made it easier for them to provide physical care; however, skilled proactive AE prescription and training by occupational and physiotherapists in the use of this equipment enhanced their confidence and capacity to care. Importantly, the caring relationship is complex and dying takes place within pre-existing relationship dynamics which can also influence the uptake of AE. The potential dangers of caregiver physical injury, particularly as many caregivers are elderly themselves, is an area which requires urgent research.

## Figures and Tables

**Table 1:** Participant demographics.

**Sex**	Patients: Female (10), Male (4)
	Caregivers: Female (2 *), Male (9)
**Ages**	Range: 55–77 years
**Living arrangements**	Two participants lived alone, the remainder lived with one or more family members
**Caregiver role**	Husband (8), wife (1), daughter (1), son (1)
**Patient diagnoses**	Lung, CNS, breast, prostate, renal, colorectal and haematological cancers, COPD

* One participant was the caregiver for his wife who had cognitive deficits post-surgery for a cerebral aneurysm.

**Table 2:** Themes and subthemes.

Theme	Sub-Themes	
3.1. Enabling engagement in everyday activities	*3.1.1. It’s been brilliant. I can get* *out!*	*3.1.2. I feel more secure and* *confident.*
3.2. Dependency: A two-way street	*3.2.1. Minimising dependency on* *caregivers*	*3.2.2. Ways that caregivers* *manage dependency*
3.3. The pragmatics of choosing, using or declining AE	*3.3.1. If you need it, you need it*	*3.3.2. I’m not that old yet…* *maybe one day*

## Data Availability

Data can be provided on request to bona fide researchers.
